# The Frequency and Associated Factors of Asymmetrical Prominent Veins: A Predictor of Unfavorable Outcomes in Patients with Acute Ischemic Stroke

**DOI:** 10.1155/2021/9733926

**Published:** 2021-09-17

**Authors:** Yue Wang, Jingjing Xiao, Li Zhao, Shaoshi Wang, Mingming Wang, Yu Luo, Huazheng Liang, Lingjing Jin

**Affiliations:** ^1^Department of Neurological Rehabilitation, Shanghai YangZhi Rehabilitation Hospital (Shanghai Sunshine Rehabilitation Center), School of Medicine, Tongji University, Shanghai 201619, China; ^2^Department of Neurology, Shanghai Fourth People's Hospital, School of Medicine, Tongji University, Shanghai, China; ^3^Translational Research Institute of Brain and Brain-Like Intelligence Affiliated to Tongji University School of Medicine, Shanghai, China; ^4^Administration Department of Nosocomial Infection Affiliated to Zhongshan Hospital of Dalian University, China; ^5^Department of Radiology, Shanghai Fourth People's Hospital, School of Medicine, Tongji University, Shanghai, China; ^6^Neurotoxin Research Center of Key Laboratory of Spine and Spinal Cord Injury Repair and Regeneration of Ministry of Education, Tongji University School of Medicine, 389 Xincun Road, 200065 Shanghai, China

## Abstract

**Objectives:**

The present study is aimed at investigating the frequency and associated factors of asymmetrical prominent veins (APV) in patients with acute ischemic stroke (AIS).

**Methods:**

Consecutive patients with AIS admitted to the Comprehensive Stroke Center of Shanghai Fourth People's Hospital between January 2013 and December 2017 were enrolled. MRI including diffusion-weighted imaging (DWI), perfusion-weighted imaging (PWI), and susceptibility-weighted imaging (SWI) was performed within 12 hours of symptom onset. The volume of asymmetrical prominent veins (APV) was evaluated using the Signal Processing In nuclear magnetic resonance software (SPIN, Detroit, Michigan, USA). Multivariate analysis was used to assess relationships between APV findings and medical history, clinical variables as well as cardio-metabolic indices.

**Results:**

Seventy-six patients met the inclusion criteria. The frequency of APV ≥ 10 mL was 46.05% (35/76). Multivariate analyses showed that proximal artery stenosis or occlusion (≥50%) (*P* < 0.001, adjusted odds ratio (OR) = 660.0, 95%CI = 57.28-7604.88) and history of atrial fibrillation (*P* < 0.001, adjusted OR = 10.48, 95%CI = 1.78-61.68) were independent factors associated with high APV (≥10 mL).

**Conclusion:**

Our findings suggest that the frequency of APV ≥ 10 mL is high in patients with AIS within 12 hours of symptom onset. History of atrial fibrillation and severe proximal artery stenosis or occlusion are strong predictors of high APV as calculated by SPIN on the SWI map.

## 1. Introduction

Susceptibility-weighted imaging (SWI) has been increasingly used to observe asymmetrical prominent veins (APV) in the cerebral cortex and deep brain structures in patients with acute ischemic stroke (AIS) [1–6]. It is generally believed that APV includes asymmetrical prominent cortical veins (APCV) and asymmetrical deep medullary veins (ADMV) [7]. The presence of ADMV is related to APCV on SWI [7].

Subsequent studies have shown that APV is associated with the prognosis of AIS patients. A recent study on 43 patients with AIS demonstrated that APV was an independent prognostic factor for clinical outcome at 3 months [5]. Another study on 61 patients treated with recombinant tissue plasminogen activator reported that multiple hypointense vessels on SWI could predict early neurological deterioration [1]. A recent study on 145 patients with AIS showed that APV might be a useful predictor for poor outcome at 3 months [2].

APV was typically defined as (1) the diameter of veins in the ischemic hemisphere was larger than that of the contralateral side and/or (2) the length and the number of veins in the ischemic hemisphere were increased compared to the contralateral hemisphere [1, 5, 8–10]. The presence of APV has been proposed to be related to increased deoxyhemoglobin which is closely related to the reduction of oxygen saturation and increase of oxygen extraction fraction [11–13].

It has been hypothesized that the ischemic area with APV represents the penumbra in ischemic stroke [11, 14, 15]. Recent studies have also shown that the APV is correlated with hypoperfusion [5, 16, 17]. A recent study on AIS patients with severe intracranial arterial stenosis or occlusion reported a 55% (60/109) prevalence of APV on SWI, which suggested that vascular stenosis or occlusion might be related to APV [3]. In clinical practice, we have noted that APV often occurs in patients with larger infarction [5]. Additionally, patients' clinical symptoms might be related to the APV volume. However, there are few studies on the frequency and associated factors of APV in AIS patients. Therefore, the present study is aimed at assessing the frequency and associated factors in AIS patients.

## 2. Materials and Methods

### 2.1. Design, Setting, and Participants

This retrospective study was carried out in the Stroke Center of Shanghai Fourth People's Hospital between 1^st^ January 2013 and 31st December 2017. The Institutional Review Board of Shanghai Fourth People's Hospital approved the present study. Written informed consent was obtained from all subjects. The inclusion criteria were (1) >18 years old; (2) patients presented with ischemic stroke and diagnosed by two certified stroke neurologists using Chinese guidelines for diagnosis and treatment of acute ischemic stroke 2018; and (3) MRI scans included diffusion-weighted imaging (DWI), perfusion-weighted imaging (PWI), and SWI within 12 hours after symptom onset. The exclusion criteria were (1) imaging data were unavailable or clinical data were incomplete and (2) being pregnant or lactating. Multimodality MRI examination was completed at the same time as intravenous thrombolysis before initiating intravascular thrombectomy. A total of 76 patients (56 males and 20 females) were included in the present study.

Demographic information including age and vascular risk factors such as hypertension, diabetes mellitus, atrial fibrillation (AF), smoking, and drinking histories were recorded. Neurologic impairment on admission was assessed using the National Institute of Health Stroke Scale (NIHSS). Neurological deterioration (ND) on day 14 was evaluated as poor outcome. The definition of ND in our study refers to neurological deterioration with an increase in the NIHSS score ≥ 2 points on day 14 after admission [18]. The NIHSS and outcome were assessed by two experienced stroke neurologists who were blind to the imaging results.

### 2.2. MR Imaging Protocol

Multimodal MRI was performed on a 1.5 T scanner (MAGNETOM Avanto, Siemens Healthcare, Germany) using a standard 8-channel head coil. The scan protocol included T1-weighted images (T1WI), T2-weighted images (T2WI), fluid-attenuated inversion recovery (FLAIR), DWI, SWI, time-of-flight MR angiography (TOF-MRA), and PWI. Imaging parameters were listed below. DWI: TR = 3600 ms, TE = 102 ms, *b* value = 0 and 1000 s/mm^2^, acquisition matrix = 192 × 192, FOV = 230 mm, section thickness = 5.5 mm, section gap = 1 mm, and duration = 70 s. PWI = TR/TE = 1590/32 ms, acquisition matrix = 128 × 128, dynamic scans = 50, FOV = 230 mm, section thickness = 5 mm, section gap = 1.5 mm, and duration = 84 s. Gadopentetate dimeglumine contrast agent (Shanghai Pharmaceutical Corporation, Shanghai, China) was intravenously injected (0.2 mmol/kg body weight) at a flow rate of 4 mL/s after flushing with 30 mL saline. SWI = 3D multiecho T2∗−weighted gradient − echo sequence, TR/TE/flip angle = 49 ms/40 ms/15°, FOV = 230 mm, acquisition matrix = 221 × 320, section thickness = 1.6 mm, and duration = 351 s. TOF − MRA = TR/TE/flip angle = 25 ms/7 ms/25°, acquisition matrix = 241 × 256, section thickness = 0.7 mm, 1 slab, and duration = 189 s. The entire duration of the MR imaging protocol was 16 min. Both SWI and TOF-MRA were performed precontrast.

### 2.3. Radiologic Assessment

Original diffusion and perfusion imaging was postprocessed with an automated Rapid Processing of Perfusion and Diffusion (RAPID) software (Ischemaview USA, Version 4.9). The infarct lesion was measured by using the volume of the infarct core on ADC (ADC < 0.62 × 10^3^ mm^2^/s) and DWI (bright sign) maps. Meanwhile, DWI volume corresponds to ADC < 0.62 × 10^3^ mm^2^/s. The hypoperfused lesion (the volume of PWI) was defined as the volume of time-to-maximum of the residue function (Tmax) delay > 6 s [19]. By using the arterial input function of the contralateral middle cerebral artery, the Tmax graphs were generated by deconvolving of the tissue concentration-time curve.

The MRA was independently rated by 2 experienced neuroradiologists. Artifactual lesion areas were visually identified by an experienced stroke neurologist (Y.W.) and a neuroradiologist (Y.L.).

### 2.4. Definition of the APV

**Figure 1:**
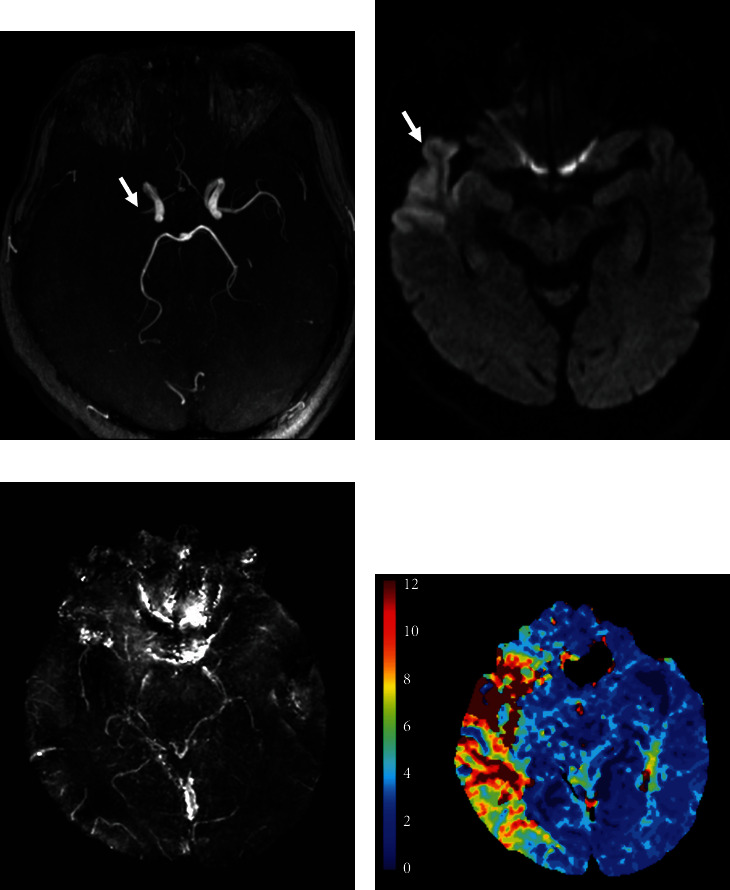
Examples of asymmetrical prominent veins on SWIM with corresponding DWI and time to maximum (Tmax) maps. (a) MR angiography showed occlusion of the right middle cerebral artery. (b) DWI showed a few small acute infarction lesions. (c) SWIM showed the APV region (yellow), which extended beyond the infarct core. The white arrow pointed to the thrombus. (d) Tmax > 6 s map (red and yellow).

The processing included the following steps: firstly, the Susceptibility Mapping and phase Artifacts Removal Toolbox (SMART; Detroit, MI, USA) software was used to reconstruct the SWI mapping (SWIM) and maximum intensity projections (MIPs, slice thickness = 7 mm) [20]. Secondly, the Signal Processing In nuclear magnetic resonance (SPIN; Detroit, Michigan, USA) software was used to analyze and measure the APV volume on SWIM [8]. Thirdly, a threshold of the mean susceptibility value plus two times the standard deviation of the veins from the contralateral hemisphere was applied to remove the background brain tissue [8]. Fourthly, the final volume of APV of each participant was calculated using the Cavalieri method with the slice thickness and gap [8, 20] (see the Supplementary materials for details (available here)).  Figure 1 showed an example of MRA, DWI, APV, and PWI.

All assessments were independently performed by two neuroradiologists (with 7 years and 10 years of MRI experience, respectively) who were blind to clinical data and other MR results. If their results were consistent, the final volume of APV for each participant was recorded as the mean of two individual values. If results from these two neuroradiologists were inconsistent, a third expert would be invited to work on this until a consensus was reached. The volume value of patients with negative APV was marked as 0 mL. Based on clinical research results, a threshold of 10 mL for APV was optimal to distinguish the high (≥10 mL) risk group from the low (<10 mL) risk group [21–26].

## 3. Statistical Analysis

Continuous variables were expressed as mean ± SD or median and interquartile range; categorical variables were presented as percentages. Interrater reliability of APV was assessed by using intraclass correlation coefficient (ICC) statistics. Clinical and imaging variables between low and high APV groups were analyzed by using *t*-test or Mann–Whitney *U*-test for continuous variables and Pearson chi-square test or Fisher's exact test for categorical variables. All participants were divided into two groups with regard to the volume of APV (APV < 10 mL as normal and APV ≥ 10 mL as abnormal). Univariate and multivariate binary logistic regression models were used to screen independent contributing factors to APV. Specific variables (age, sex, NIHSS, and DWI) were preselected for entry into the model. Other possible variables were selected for entry into the model if *P* ≤ 0.05 in the univariate analysis. Relationships between probably associated variables were examined by calculating Spearman correlation coefficients for continuous data or Kendall's tau-*b* for categorical data. Independent risk factors of APV were analyzed using multivariate logistic regression analysis. All association data were expressed as OR with corresponding 95% confidence intervals (CI) and *P* values. Statistical significance was defined as *P* < 0.05 (two sided). All data were analyzed using SPSS (version 20.0) for Windows (SPSS Inc., Chicago, IL, USA).

## 4. Results

**Figure 2:**
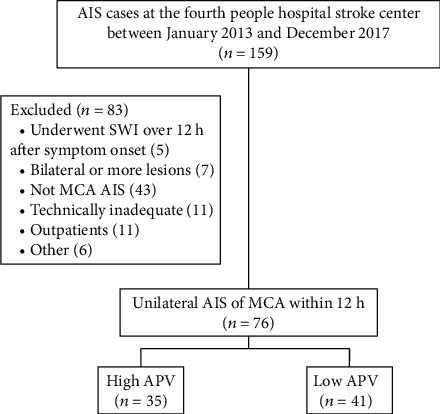
Flow chart of patient recruitment. AIS: acute ischemic stroke; SWI: susceptibility weighted imaging; MCA: middle cerebral artery; APV: asymmetrical prominent cortical veins.

A total of 159 patients were screened for AIS within 12 h after onset at the Stroke Center of Shanghai Fourth People's Hospital Affiliated to Tongji University School of Medicine, between January 2013 and December 2017. Seventy-six patients met the inclusion criteria. Eighty-three patients were excluded; among them, 43 had lesions not in the territory of MCA, 7 patients had bilateral lesions or more, 5 completed SWI over 12 h after symptom onset, 11 had inadequate information, and 17 had other issues (Figure 2).

### 4.1. Patient Characteristics

A total of 76 subjects, including 56 males and 20 females, were included in the study. The mean age of patients was 70.07 ± 1.37 years in the range of 37 to 94. The median time between SWI and symptom onset was 3.5 h (interquartile range (IQR): 2–7 h).

The median (IQR) NIHSS on admission was 6 (2–12). The median (IQR) DWI volume was 2 mL (1–20) (Table 1).

Table 1 presented the sociodemographic characteristics and clinical risk factors associated with different APV volumes. There was significant difference in history of AF (*P* < 0.001), admission NIHSS (*P* = 0 .001), DWI volumes (*P* < 0.01), PWI volumes (*P* < 0.001), and stenosis (50%) (*P* < 0.001) between patients with low (APV < 10 mL) and high APV volumes (APV ≥ 10 mL). Patients with high APV tended to have poorer outcome than those with low APV 14 days later (*P* = 0.031).

### 4.2. Interrater Agreement for Evaluation of Prominent APV

Interrater agreement for the evaluation of APV volume was excellent (ICC = 0.995). High APV (APV ≥ 10 mL) was seen in 35/76 (46.05%) patients (mean: 116 mL; range: 35-271 mL).

### 4.3. Factors Associated with High APV

In univariate binary logistic regression analysis, history of AF (*P* < 0.001, OR = 16.89, 95%CI = 4.37–65.32), baseline NIHSS score (*P* < 0.001, OR = 1.14, 95%CI = 1.05–1.23), time to imaging (h) (*P* < 0.001, OR = 0.81, 95%CI = 0.70–0.94), DWI volume (mL) (*P* < 0.01, OR = 0.81, 95%CI = 1.10–1.17), and symptomatic stenosis (50%) (*P* < 0.001, OR = 660.0, 95%CI = 57.28–7604.88) were significantly associated with high APV after ischemic stroke (Table 2). There was a positive correlation between history of AF and stenosis (50%) (*r* = 0.62, *P* < 0.001) and baseline NIHSS and DWI volume (*r* = 0.55, *P* < 0.001) in all patients.

Multivariate logistic regression modeling was performed for independent predictors with *P* < 0.05 in the univariate analysis and without significant correlation. In the multivariate logistic regression analysis, history of atrial fibrillation was significantly associated with high APV (OR: 10.48; 95% CI: 1.78-61.68) after adjusting for history of AF, baseline NIHSS, and time to imaging (h) (model 1). According to the MR imaging data, stenosis (50%) was significantly associated with high APV (OR: 660.0; 95% CI: 57.28-7604.88) after adjusting for time to imaging (h), DWI volume, and stenosis (50%) (model 2) (Table 2).

### 4.4. Correlation between Hypoperfusion and APV

**Figure 3:**
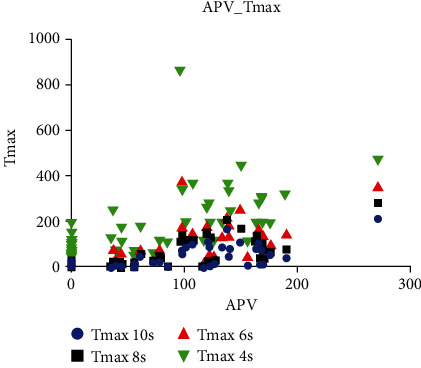
Correlation between Tmax and APV.

Time to maximum (Tmax) maps were automatically produced using the RAPID software. Volumes of hypoperfusion based on different Tmax map thresholds (Tmax > 4 s, Tmax > 6 s, Tmax > 8 s, and Tmax > 10 s) were calculated. The volume of Tmax > 6 s is the most commonly used one representing ischemic penumbra [27–29]. There was a significant positive correlation between APV and Tmax > 6 s (*r* = 0.865, *P* < 0.001) and between APV and Tmax > 8 s (*r* = 0.845, *P* < 0.001) (Table 3 and Figure 3).

## 5. Discussion

To our best knowledge, this is the first study to report both the frequency and associated risk factors of asymmetrical prominent veins in AIS patients within 12 hours after symptom onset. The frequency of high APV (volume ≥ 10 mL) was 46.05% (35/76). Furthermore, we found that history of AF and DWI volume were independent factors associated with high APV.

### 5.1. The Frequency of MR Perfusion Abnormality

Our study showed a 46.05% (95% CI: 35.31-57.17%) frequency of high asymmetrical prominent veins (APV ≥ 10 mL) in AIS patients within 12 hours. This is higher than that of a previous report which showed a prevalence of 24.14% (35/145)^2^, but lower than that of the other two studies whose prevalence was 55.05% (60/109) ^3^ and 60.78% (31/51)^4^, respectively. There are a few possible reasons for this difference. First, the variability of results in these studies is related to the inconsistent definition of asymmetrical prominent veins. In this study, APV included asymmetric deep medullary veins (ADMV) and asymmetric prominent cortical veins (APCV). In other studies, the prominent asymmetric veins predominantly include APCV. Second, the present study calculated quantitative volumes of APV using a semiautomatic software, while other studies categorized APV using manual evaluation. In two recent studies, APCV was defined as a regional prominence of low-intensity vessels with increased vessel number and/or diameter on the ipsilateral side than on the contralateral hemisphere [2, 3]. Meanwhile, APCV was divided into two categories (positive and negative) ^3^ and four grades (0, normal appearance of cortical veins; 1, slight; 2, moderate; and 3, distinct) ^2^, respectively. Third, the asymmetrical prominent veins of the subjects were related to the time of onset and the time of SWI completion. In this study, SWI scan was completed within 12 hours after stroke onset for all AIS patients. The median interval between SWI completion and symptom onset was 3.5 (interquartile range (IQR): 2–7) hours. In a recent study, brain MRI was performed within 72 hours after ischemic stroke onset with a median time of 22 (range: 8–58) hours^2^. In another study, SWI was performed within 3 days after stroke onset with a mean over 30 (34.4 ± 27.36 in APCV positive group; 39.80 ± 28.87 in APCV negative group) hours ^4^.

### 5.2. Risk Factors Associated with APV

The initial observation by Xia et al. [8] in 2014 of asymmetrically prominent cortical veins in the ipsilateral hemisphere of AIS participants was quantified by comparing with contralateral changes in oxygen extraction fraction (OEF) [30]. Subsequent studies have shown a strong relationship between the presence of APV and early prognosis in patients with acute stroke [1–3, 5]. SWI is very sensitive for the detection of susceptibility differences [30]. After AIS, the venous blood volume increases as a result of vasodilation due to increase in oxygen extraction [31] and in the venous deoxyhemoglobin concentration [12, 13]. APV may appear on SWI due to differences in the concentrations of deoxyhemoglobin between cerebral veins and the surrounding parenchyma caused by cerebral vascular occlusion.

There are a number of possible clinical risk factors for APV in patients with AIS. Our first hypothesis, based on results of previous studies, was that clinical manifestations and vascular risk factors were associated with APV in patients with AIS [1, 4, 7]. Of the 76 stroke subjects included in this study, 30.3% had a history of AF, which is similar to a previous report in China which showed a prevalence of 20.2% (305/1511) among patients with ischemic stroke and TIA [32]. Among 35 patients with APV ≥ 10 mL after acute stroke onset, 57.14% had a history of AF. The high APV group completed the SWI examination about 2 hours earlier than the low APV group. According to results of model 1 in multifactor analysis, history of AF (OR: 10.48; 95% CI: 1.78-61.68; *P* < 0.001) and time to imaging (h) (OR: 0.81; 95% CI: 0.74-0.99; *P* = 0.038) were independent risk factors of high APV with ischemic stroke. In the present study, the baseline NIHSS was not associated with high APV. However, there was a strong positive correlation between baseline NIHSS and DWI volume (*r* = 0.55, *P* < 0.001) in patients with AIS. Therefore, these findings suggest that the earlier the examination was completed, the more likely the AIS patients with a history of AF were to have high APV within 12 hours of onset.

Our second hypothesis was that MR imaging was associated with high APV in AIS patients. Among 76 stroke patients included in this study, 44.7% had proximal artery stenosis or occlusion (≥50%), which is similar to that of a previous study [1, 5]. In the present study, 33 of 35 patients with APV ≥ 10 mL after acute stroke onset had proximal artery stenosis or occlusion (≥50%). These findings support that proximal artery stenosis or occlusion (≥50%) was an independent risk factor of high APV in AIS patients (OR: 660.0; 95% CI: 57.28-7604.88; *P* < 0.001) as shown in model 2 using the multiple logistic regression analysis. In this study, it was found that the DWI volume was not an independent risk factor of high APV using the same method.

The present study showed a strong relationship between the presence of proximal artery stenosis or occlusion (≥50%) and asymmetrical prominent veins in AIS patients within 12 hours. Causes of acute proximal artery stenosis or occlusion were listed below. First, embolization can cause stenosis of large blood vessels in patients with atrial fibrillation [33]. Second, rupture of large atherosclerotic plaques leads to insufficient blood supply to the distal end due to thrombosis after exposing highly thrombogenic, red cell-rich necrotic core material [34]. Third, thrombi formed on lesions (plaque erosion) without rupture render pathological intimal thickening or fibroatheromas [34]. Vascular stenosis or blockage results in decrease in SPO_2_ in both arteries and veins in the ischemic area [4] and increase in OEF [30], which consequently increased paramagnetic substances (deoxyhemoglobin) in blood vessels. As a result, the appearance of APV was observed on SWI [20, 30]. Our research showed that there was a significant positive correlation between low perfusion (Tmax > 6 s, *r* = 0.865, *P* < 0.001; Tmax > 8 s, *r* = 0.845, *P* < 0.001) and APV, which is consistent with findings of a recent study [26].

The current study has a couple of limitations. First, it is a crosssectional study with a relatively small sample size (*n* = 76). Therefore, it cannot demonstrate direct causality between risk factors and APV in AIS patients. Second, the present study lacks follow-up results. It is unknown whether APV observed in our study progressed or disappeared after initial imaging. Therefore, findings of this study should be considered preliminary and be further verified in the future.

## 6. Conclusions

In conclusion, history of atrial fibrillation and proximal artery stenosis or occlusion (≥50%) are strong predictors of asymmetrical prominent veins observed on SWI maps of AIS patients within 12 hours of symptom onset.

## Figures and Tables

**Table 1:** Clinical and imaging characteristics of AIS patients with low or high APV.

Characteristics	Total (*n* = 76)	Low 0-10 (*n* = 41)	High ≥10 (*n* = 35)	*P* value
Age (yr)	70.04 ± 1.37	67.85 ± 12.71	72.6 ± 10.58	0.087
Sex, male	56 (73.7)	28 (68.3)	28 (80.0)	0.251
Medical history				
Hypertension	56 (73.7)	33 (80.5)	23 (65.71)	0.149
Diabetes mellitus	26 (34.2)	17 (41.5)	9 (25.71)	0.152
Atrial fibrillation	23 (30.3)	3 (7.3)	20 (57.14)	0.000
Prior TIA or stroke	12 (15.8)	7 (17.1)	5 (14.29)	0.740
Smoking	27 (35.5)	17 (41.5)	10 (28.57)	0.244
Drinking	14 (18.4)	8 (19.5)	6 (17.14)	0.791
Ghb (g/L)	135.72 ± 2.01	138.34 ± 15.46	132.66 ± 19.45	0.162
FBG (mmol/L)	6.05 (5.03-7.95)	6.0 (4.9-7.7)	6.1 (5.1-8.1)	0.528
LDL (mmol/L)	2.71 (2.03-3.43)	2.05 (2.85-3.53)	2.64 (2.01-3.29)	0.418
Baseline NIHSS score	6 (2-12)	5 (2-9)	10 (10-25)	0.001
Time to imaging (h)	3.5 (2-7)	5 (2.25-10)	3 (1-5)	0.005
DWI volume (mL)	2 (1-20)	1 (1-3.5)	20 (1-33)	0.002
PWI volume (mL)	10.5 (0-96.25)	0 (0-1.5)	137 (49-154)	0.001
Stenosis (50%)	34 (44.7)	1 (2.4)	33 (94.29)	0.000
Poor outcome	27 (35.5)	10 (24.4)	17 (48.6)	0.031

Table cells express results in mean ± SD for normally distributed continuous variables, *n* (%) for dichotomous variables, and median (interquartile range) for ordinal variables and nonnormally distributed continuous variables, respectively. The DWI volume of the infarct core has been acquired on ADC maps (ADC < 0.62 × 10^3^ mm^2^/s) and the volume of PWI on Tmax > 6 s maps.

**Table 2:** Factors associated with high volume of asymmetrical prominent veins.

Variable	Univariate analysis		Model 1		Model 2	
	Crude OR (95% CI)	*P* value	Adjusted OR (95% CI)	*P* value	Adjusted OR (95% CI)	*P* value
Atrial fibrillation	16.89 (4.37-65.32)	0.000	10.48 (1.78-61.68)	0.009		
Baseline NIHSS score	1.14 (1.05-1.23)	0.001	1.03 (0.92-1.16)	1.033		
Time to imaging (h)	0.81 (0.70-0.94)	0.005	0.84 (0.71-0.99)	0.038	0.79 (0.53-1.18)	0.247
DWI volume (mL)	1.10 (1.04-1.17)	0.002			1.06 (0.96-1.18)	0.271
Stenosis (50%)	660.0 (57.28-7604.88)	0.000			432.14 (30.93-6038.35)	0.000

Model 1: based on clinical inquiry and physical examination. Adjusted for history of atrial fibrillation, baseline NIHSS, and time to imaging (h). Model 2: based on relevant MR imaging data. Adjusted for time to imaging (h), DWI volume, and stenosis (50%).

**Table 3:** Correlation between Tmax and APV.

Tmax	APV	
	*r*	*P* value
Tmax > 4 s	0.785	*P* < 0.001
Tmax > 6 s	0.864	*P* < 0.001
Tmax > 8 s	0.845	*P* < 0.001
Tmax > 10 s	0.801	*P* < 0.001

## Data Availability

Data produced for this manuscript are available from the corresponding author upon reasonable request.

## Supplementary Materials

Supplementary Materials Supplementary file 1: supplementary table 1 showing MR imaging parameters. Supplementary file 2: APV volume measurement.
