# Supplementary material for: The Frequency and Associated Factors of Asymmetrical Prominent Veins: A Predictor of Unfavorable Outcomes in Patients with Acute Ischemic Stroke
Source: Neural Plast. 2021 Sep 17;2021:9733926. doi: 10.1155/2021/9733926 (PMC8463180; doi:10.1155/2021/9733926)
Supplement: Supplementary Materials — Supplementary file 1: supplementary table 1 showing MR imaging parameters. Supplementary file 2: APV volume measurement. [file 9733926.f1.docx]

**SUPPLEMENTARY MATERIALS**

**MATERIALS AND METHODS**

**Supplementary file 1.**

**Supplementary Table 1. MR Imaging Parameters**

| MRI  protocols | FOV  (mm×mm) | Matrix Size | Slices | Slice Thick  -ness  (mm) | TR/TE  (msec) | Flip Angle  (degree) | Bandwidth  Hz/Px | Scan time  (sec) |
| --- | --- | --- | --- | --- | --- | --- | --- | --- |
| T1WI | 230×230 | 186×256 | 18 | 5.5 | 400/8.7 | 90 | 150 | 78 |
| T2WI | 230×230 | 261×384 | 18 | 5.5 | 3800/91 | 150 | 191 | 70 |
| FLAIR | 230×230 | 256×190 | 18 | 5 | 4000/92 | 150 | 190 | 114 |
| DWI | 230×230 | 192×192 | 19 | 5.5 | 3600/102 | / | 964 | 70 |
| TOF-MRA | 180×180 | 241×256 | 64 | 0.7 | 25/7 | 25 | 100 | 189 |
| SWI | 230×230 | 221×320 | 72 | 1.6 | 49/40 | 15 | 80 | 351 |
| PWI | 230×230 | 128×128 | 19 | 5 | 1590/32 | 90 | 1346 | 84 |
|  | | | | | | | | |

**Supplementary file 2.**

**APV volume measurement**

Firstly, the assessment of APVs on SWIM were relatively objective through quantitative analysis by SPIN (signal processing in nuclear magnetic resonance) software according to the measurement method of a previous study (reference: Xia S, Utriainen D, Tang J, et al (2014) Decreased oxygen saturation in asymmetrically prominent cortical veins in patients with cerebral ischemic stroke. Magn Reson Imaging 32:1272-1276.). The detailed process was as follows (Fig.1-Fig.4, SWIM image of a 70-years-old male with acute ischemic stroke and typical APVs in the right cerebral hemisphere)：


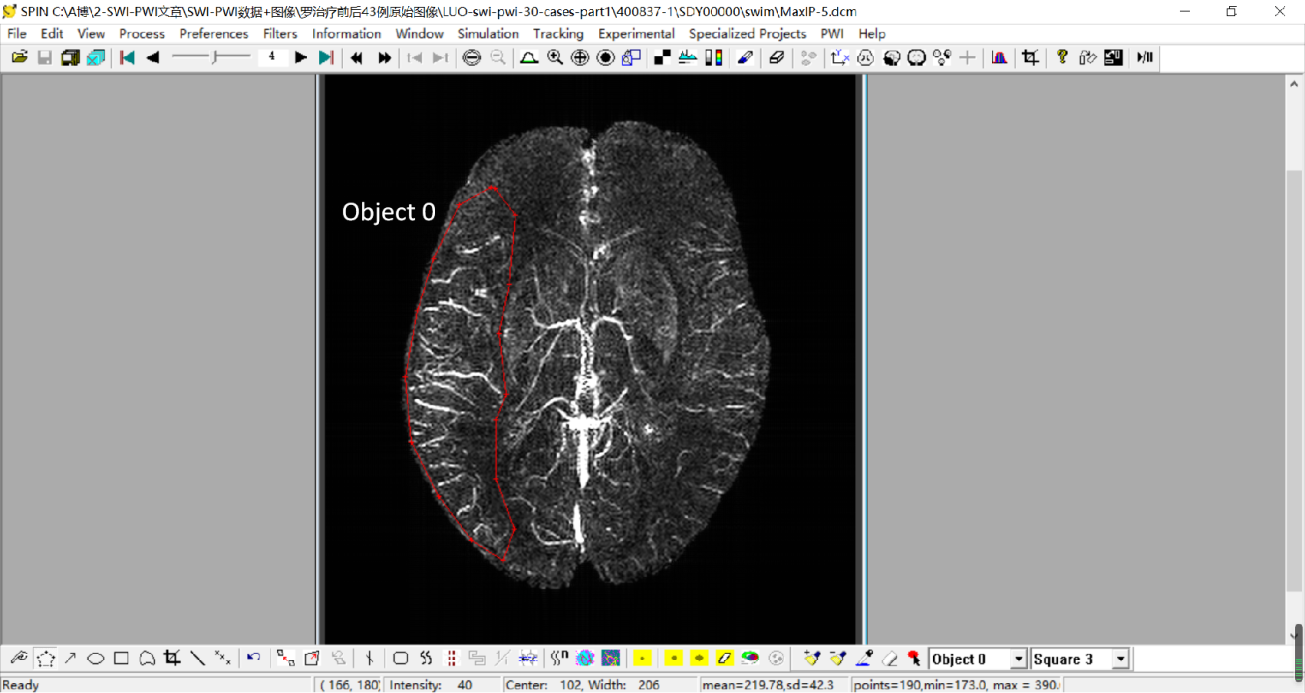


Fig.1 Demonstration of the selection of cortical veins in ischemic hemisphere on SWIM (Object 0). The original SWIM shows higher intensity in the right ischemic hemisphere.


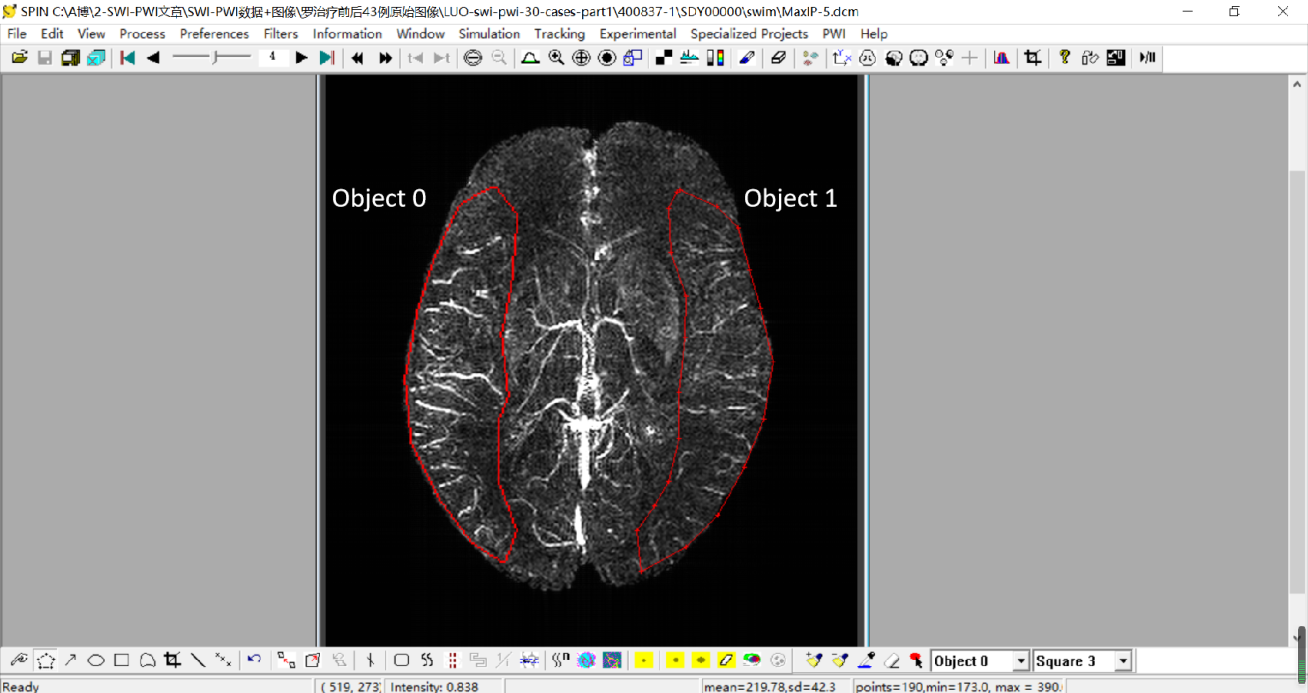


Fig.2 Demonstration of the selection of cortical veins in control left hemisphere on SWIM (Object 1). While the control left hemisphere still shows visible structures with lower susceptibility.


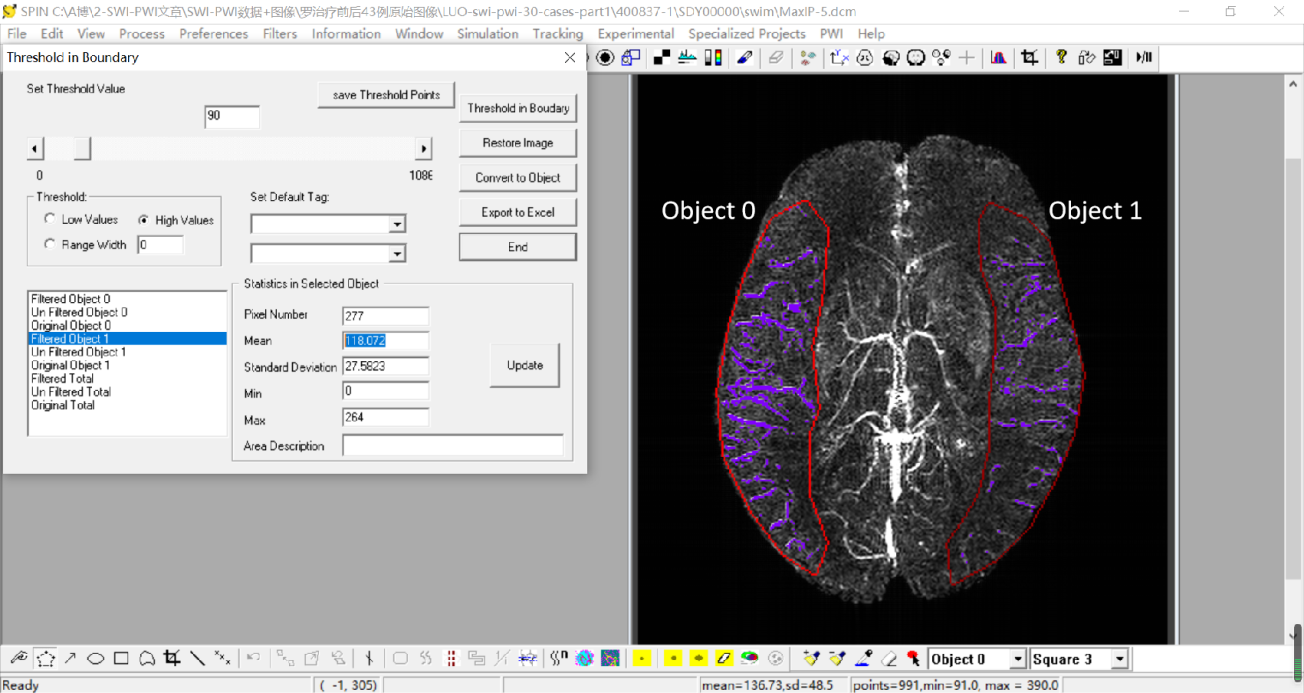


Fig.3 The lower threshold of 90 ppb (green frame) is applied to the SWIM data to highlight the cortical veins on the control side (Object 1) and determine the mean and standard deviation of the vein’s susceptibility (red frame). This value is chosen because it is the lower end of susceptibility values for cortical veins in the control and, therefore, it removes most of the background tissue while preserving the signal from the cortical veins to be included in the analysis.


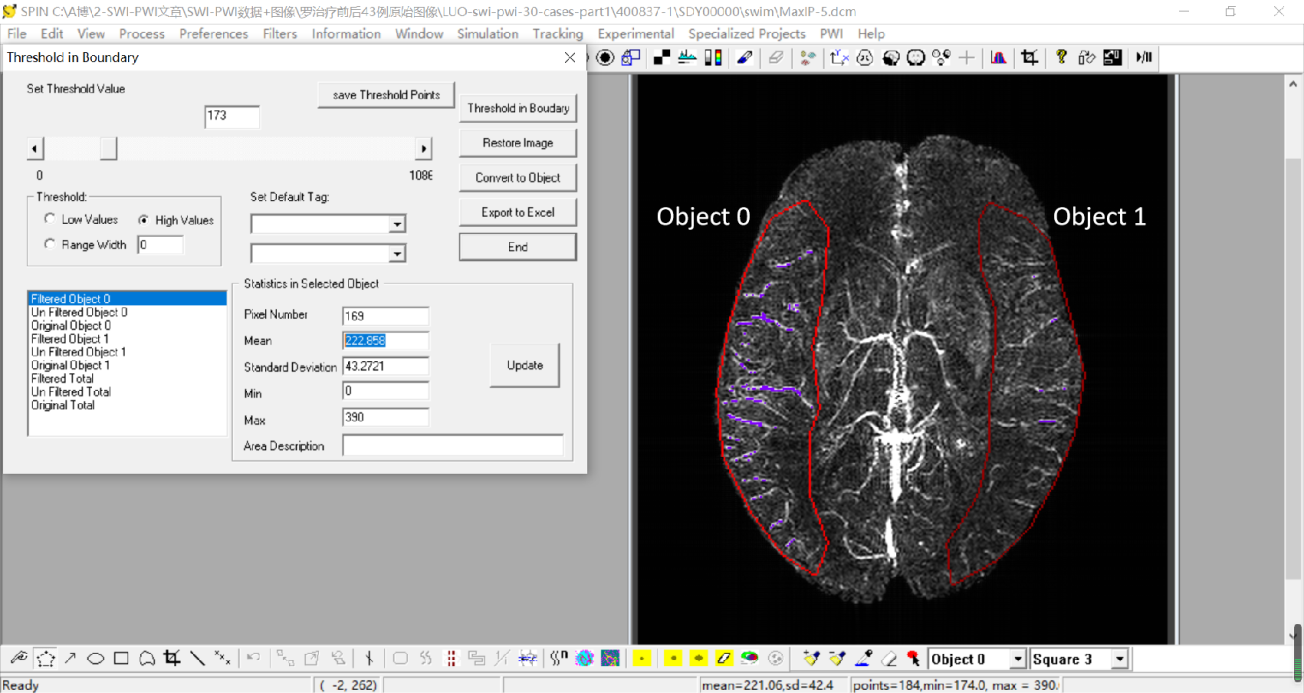


Fig.4 The mean value ± two times standard deviation of the susceptibility of the veins in the control hemisphere (green frame) is used to establish the upper threshold to extract abnormal veins (i.e., APV). Note that the visibility of venous structures is increased in the ischemic hemisphere (Object 0). So we can clearly identify the edges of dilated cortical vessels.

Secondly, patients with ischemic stroke often have APVs with high susceptibility values, indicating high OEF in the local tissue drained by these veins (Haacke et al., 2010; Xia et al., 2014.) It is believed that the brain tissue in the APVs distribution area is hypoxic in patients with ischemic stroke. At the same time, the same mechanism exists for deep veins which present with larger diameters and/or increased length compared to those in the contralateral hemisphere, caused by increased deoxyhemoglobin in veins (Fan et al., 2020). So if the deep veins are dilated, they should also be within the delineated range (Fig.5 ).


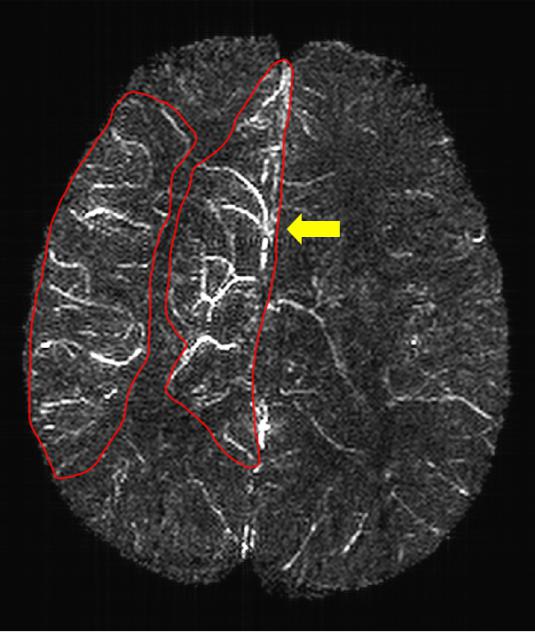


**Fig.5 The yellow thick arrow indicates the dilated deep veins.**

Thirdly, in our study, when drawing the ROI of APCVs on SWIM images, we selected the edge of each prominent hyperintense vein near the side of the cerebral cortex as the boundary on every slice. Points manually drawn along the edge of each prominent cortical vein were automatically connected to form a closed ROI (Fig.6 red lines represent the the ROI tracing). The ROI was drawn as small as possible on the basis of ensuring that all prominent cortical veins were included. Moreover, because the mean value ± two times standard deviation of the susceptibility of the veins in the control hemisphere was used to define APVs, the background tissue including brain tissue was removed even if there was some excessive brain tissue included in the ROI.


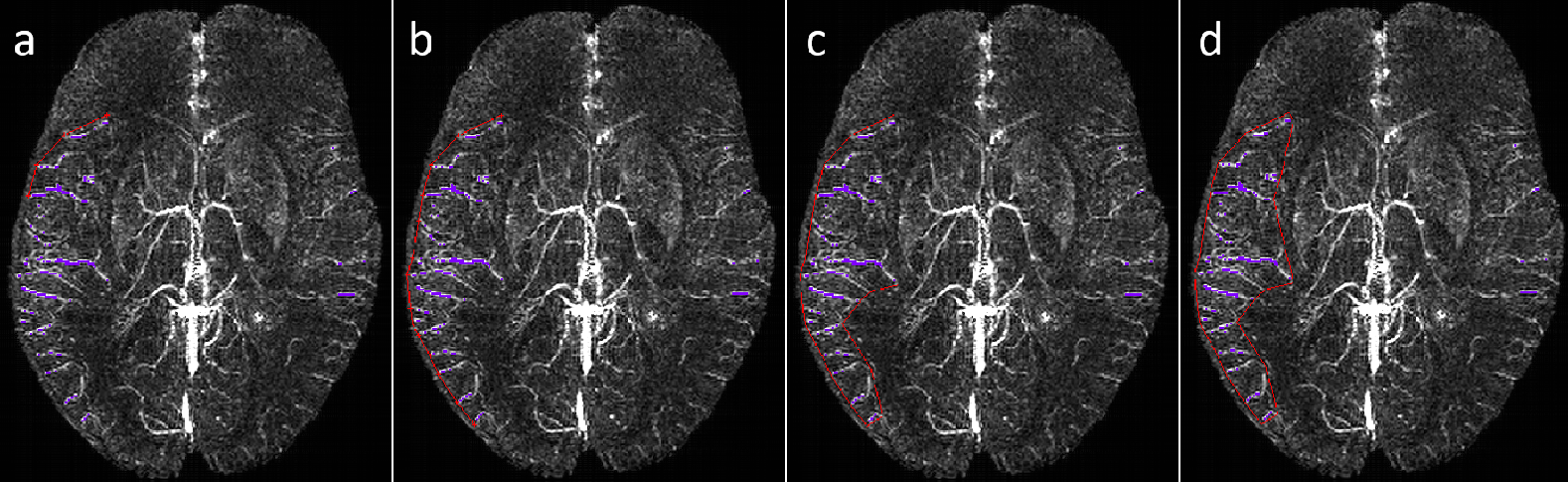


Fig.6 (a-d) Red lines represent the ROI tracing of APCVs on SWIM.

The APVs territory was manually outlined along the edge of prominent hyperintense vessels near the side of the cerebral cortex on every slice. Points manually drawn along the edge of each prominent cortical vein were automatically connected to form a closed region of interest (ROI) (Fig.6). The ROI was drawn as small as possible on the basis of ensuring that all prominent cortical veins were included. If the deep veins were dilated, they should also be within the delineated range, but only above the basal ganglia slice.
